# Screening for Human Immunodeficiency Virus, Hepatitis B Virus, Hepatitis C Virus, and Treponema pallidum by Blood Testing Using a Bio-Flash Technology-Based Algorithm before Gastrointestinal Endoscopy

**DOI:** 10.1128/JCM.00986-16

**Published:** 2016-11-23

**Authors:** Zhou Jun, Chen Zhen, Zhang QuiuLi, An YuanQi, Verónica Vocero Casado, Yuan Fan

**Affiliations:** a Department of Blood Transfusion, Beijing Military General Hospital of PLA, Beijing, China; b Marketing Department, Biokit, Lliçà d′Amunt, Barcelona, Spain; Memorial Sloan-Kettering Cancer Center

## Abstract

Currently, conventional enzyme immunoassays which use manual gold immunoassays and colloidal tests (GICTs) are used as screening tools to detect Treponema pallidum (syphilis), hepatitis B virus (HBV), hepatitis C virus (HCV), human immunodeficiency virus type 1 (HIV-1), and HIV-2 in patients undergoing surgery. The present observational, cross-sectional study compared the sensitivity, specificity, and work flow characteristics of the conventional algorithm with manual GICTs with those of a newly proposed algorithm that uses the automated Bio-Flash technology as a screening tool in patients undergoing gastrointestinal (GI) endoscopy. A total of 956 patients were examined for the presence of serological markers of infection with HIV-1/2, HCV, HBV, and T. pallidum. The proposed algorithm with the Bio-Flash technology was superior for the detection of all markers (100.0% sensitivity and specificity for detection of anti-HIV and anti-HCV antibodies, HBV surface antigen [HBsAg], and T. pallidum) compared with the conventional algorithm based on the manual method (80.0% sensitivity and 98.6% specificity for the detection of anti-HIV, 75.0% sensitivity for the detection of anti-HCV, 94.7% sensitivity for the detection of HBsAg, and 100% specificity for the detection of anti-HCV and HBsAg) in these patients. The automated Bio-Flash technology-based screening algorithm also reduced the operation time by 85.0% (205 min) per day, saving up to 24 h/week. In conclusion, the use of the newly proposed screening algorithm based on the automated Bio-Flash technology can provide an advantage over the use of conventional algorithms based on manual methods for screening for HIV, HBV, HCV, and syphilis before GI endoscopy.

## INTRODUCTION

The potential for transmission of infections during surgical procedures or gastrointestinal (GI) endoscopy (gastroscopy and enteroscopy) is a matter of concern for both physicians and patients (1–7). Currently, transmission of infections during GI endoscopy is a rare event with a transmission rate of 1 in 1.8 million endoscopy procedures (1). Further, documentation of the transmission of viral infections by GI endoscopy is difficult due to the long incubation periods of these infections and the presence of no or minimal symptoms in patients with these infections (1). Preoperative routine testing for the presence of infective pathogens may benefit these patients, as it can provide information on the patient's infection status and help with subsequent counseling, care, and access to treatment, where applicable (3, 7). Also, this type of testing can provide an opportunity for health care professionals to implement measures to prevent the transmission of infections to hospital workers involved in the surgery (1, 7).

It has been suggested that preoperative testing for infective pathogens involves a large number of investigations that are often expensive, rarely detect major abnormalities, and may cause unnecessary delays or cancellation of surgeries, leading to an increase in medicolegal liability (3). Currently in China, serological methods are used for routine screening of blood samples from patients undergoing surgery and GI endoscopy for blood-transmissible infections, including infections with Treponema pallidum (syphilis), hepatitis B virus (HBV), hepatitis C virus (HCV), human immunodeficiency virus type 1 (HIV-1), and HIV-2 (1–3, 7).

A screening algorithm defines the specific tests and testing procedures to be followed during the screening for infective pathogens in each hospital facility and helps to maintain consistent results and the use of consistent decisions for the management of patients with confirmed positive results (8). All proposed algorithms should have a high level of sensitivity and specificity for testing for blood-transmitted infections to ensure safety during surgery (9–11). The selection of appropriate algorithms and assays is a critical part of the screening program, and enzyme immunoassays (EIAs) and chemiluminescent immunoassays (CLIAs) are currently the assays that are the most frequently employed in these serological screening algorithms (9–13).

While most of the currently used screening algorithms are still based on the use of gold immunoassays and colloidal tests (GICTs), the use of algorithms based on automated chemiluminescent methods can provide several important advantages (8, 14). Among the commercially available CLIAs, the Bio-Flash technology (Biokit, Barcelona, Spain) provides automated two-step CLIAs for the qualitative measurement of HBV surface antigen (HBsAg) and antibodies against HCV, HIV-1/2, and T. pallidum in human serum or plasma as an aid to the proper serological detection of HBV, HCV, HIV-1/2, and T. pallidum (14).

The present observational cross-sectional study assessed the advantages of the newly proposed algorithm based on the Bio-Flash technology over the current GICT screening algorithm with manual colloidal selenium one-step immunoassay strips in terms of sensitivity, specificity, and work flow characteristics for screening for HIV, HBV, HCV, and syphilis in patients undergoing GI endoscopy.

## MATERIALS AND METHODS

### Study design.

This observational, cross-sectional study was conducted at the Department of Blood Transfusion, Beijing Military General Hospital of PLA, Beijing, China, between 14 and 18 April 2015 and 1 and 31 July 2015. The study was performed in accordance with national legislation and the Declaration of Helsinki (revised in 2000).

**FIG 1:**
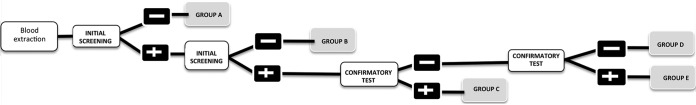
Representative screening algorithm flowchart for detection of infection markers at the Beijing Military General Hospital of PLA, Beijing, China. Positive and negative refer to positivity and negativity for any evaluated markers.

Patients undergoing GI endoscopy were invited to participate in this study. Blood samples were collected from the participating patients by venipuncture. Serum was separated from the collected blood samples and tested for HIV, HCV, HBV, and T. pallidum infection markers. Retrospective samples from patients admitted to the hospital between 8 January and 16 March 2015 were analyzed for HIV, as no HIV-positive patients enrolled in the study. A representative screening algorithm flowchart for the detection of infection markers is shown in Fig. 1.

**FIG 2:**
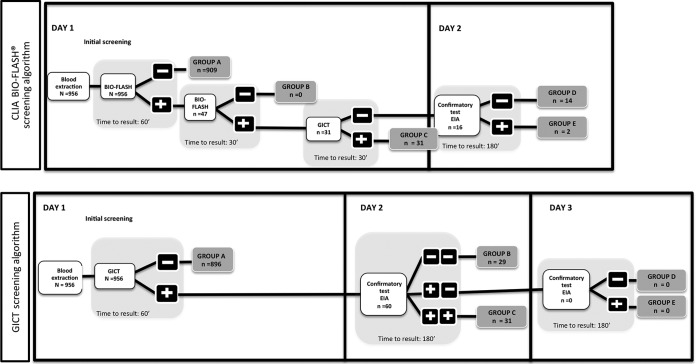
Comparison of screening algorithms and test results for the automated chemiluminescent Bio-Flash technology-based screening algorithm (the new proposed laboratory procedure) and the manual GICT screening algorithm (the regular laboratory procedure). Positive and negative refer to positivity and negativity for any evaluated markers.

The main aim of the proposed algorithm for the screening of serum samples for infection markers was the inclusion of the two-step Bio-Flash technology. Samples that were initially reactive were automatically retested using the Bio-Flash technology, and the samples reactive on retesting were subsequently analyzed by GICTs on the same day. Any samples with discordant results were analyzed on day 2 using an enzyme-linked immunosorbent assay (ELISA) (Fig. 2). In the conventional manual algorithm, the early stages of screening were performed using GICTs (as a regular laboratory procedure). Positive results were confirmed by ELISA on days 2 and 3 (Fig. 2), and the results for any samples with discordant results between ELISA and the GICTs were confirmed by Western blotting. All tests were performed per the manufacturers' instructions.

The patients were stratified into five different categories on the basis of the diagnostic test results for all markers. Group A included all patients who had negative results by the screening test, group B included all patients who had positive results by the screening test but who were confirmed to be negative in a second analysis, group C included all patients who had positive results by the screening test and who were confirmed to be positive by a second analysis, group D included all patients who had discordant results between the two early tests and who were confirmed to have a negative result by a third method, and group E included all patients who had discordant results between the two early tests and who were confirmed to have a positive result by a third method (Fig. 2).

### Automated Bio-Flash technology.

Automated two-step CLIAs were used for the qualitative measurement of IgG and IgM antibodies against HIV-1 and HIV-2 (Bio-Flash anti-HIV 1+2; Biokit, Barcelona, Spain), IgG antibodies against HCV (Bio-Flash anti-HCV; Biokit, Barcelona, Spain), IgG and IgM antibodies against T. pallidum (Bio-Flash syphilis; Biokit, Barcelona, Spain), and HBsAg (Bio-Flash HBsAg; Biokit, Barcelona, Spain) in the serum samples using the Bio-Flash instrument. The time to the first automated results was 30 min, and the ability to perform 64 tests per hour was reported after the first hour. Samples that were reactive on the initial test were automatically retested, and only samples that were reactive on retesting were considered positive.

### Manual gold immunoassays and colloidal tests.

Manual colloidal selenium one-step immunoassay strips were used for the qualitative measurement of antibodies against HIV-1 and HIV-2 (Alere Determine HIV-1/2; Alere Medical Co., Ltd., Waltham, MA, USA) and T. pallidum (Alere Determine syphilis; Alere Medical Co., Ltd., Waltham, MA, USA) in the serum samples. Individual results were visually read after 15 min for each test. Similar strips were also used for the qualitative measurement of antibodies against HCV (Ying Ke Xin Chuang [XiaMen] Technology Co., Ltd., Xiamen, China) and detection of HsBAg (Ying Ke Xin Chuang [XiaMen] Technology Co., Ltd., Xiamen, China), where individual results were visually read after 10 and 15 min, respectively.

### Enzyme-linked immunosorbent assay.

Confirmatory tests were performed using ELISAs. Two different confirmatory tests were used for each infection marker: for HIV-1/2, an antibody ELISA (Beijing Wantai Biological Pharmacy Enterprise, Beijing, China) and the GeneScreen HIV-1/2, v.2, ELISA (Bio-Rad, CA, USA); for HCV, an ELISA (Beijing Wantai Biological Pharmacy Enterprise Co., Ltd., Beijing, China) and the Murex anti-HCV, v.4.0, ELISA (Abbott Murex, Kent, UK); for T. pallidum, an EIA kit (Shanghai Kehua Bio-engineering Co., Ltd., Shanghai, China) and the syphilis EIA (Abbott Murex, Kent, UK); for HBsAg, an ELISA (Beijing Wantai Biological Pharmacy Enterprise Co., Ltd., Beijing, China) and the Murex HBsAg, v.3.0, ELISA (Abbott Murex, Kent, UK).

### Western blotting.

The results for samples with discordant results by use of the Bio-Flash technology, GICTs, and ELISAs were confirmed by Western blotting for all agents except HBV, for which the result was confirmed using five different HBV infection markers (HBeAg, anti-HBe, anti-HBc, total anti-HBc, and anti-HBs). For the detection of antibodies against HCV, an HCV antibody detection kit was used (MP Biomedical Asia Pacific Pte., Ltd., Singapore). For the detection of T. pallidum antibodies, a membrane-based test system (Euroline-WB; Euroimmun Medical Laboratory Diagnostics Stock Company, Beijing, China) and a T. pallidum-specific hemagglutination assay (Immutrep syphilis test kits; Zhuhai Xin Mei Trading Co., Ltd., Guangdong Province, China) were used. Antibodies against HIV-1/2 were detected using an MP-WB kit (HIV Blot 2.2 WB; MP Biomedicals Asia Pacific Pte., Ltd., Singapore).

### Statistical analysis.

Data were analyzed separately for each infection marker using SPSS software, v.19.0. For each test, positive and negative results were defined according to the cutoff values specified in the manufacturer's instructions. The clinical diagnosis of each infection was used as a “gold standard,” and the laboratory operators were blind to the clinical diagnosis. Test sensitivity was defined as TP/(TP + FN), where TP is the number of samples with true-positive results and FN is the number of samples with false-negative results. Similarly, test specificity was defined as TN/(TN + FP), where TN is the number of samples with true-negative results and FP is the number of samples with false-positive results. The positive predictive value (PPV) was defined as TP/(TP + FP), and the negative predictive value (NPV) was defined as TN/(TN + FN). The sensitivity, specificity, and predictive values of both algorithms were evaluated and are expressed in percent.

The level of agreement between the Bio-Flash technology and GICTs for the detection of each infection marker at an early stage of the algorithms was calculated using the kappa coefficient (κ value) and the 95% confidence interval without assumption of the null hypothesis. The laboratory work flow was evaluated in the early stages for both screening algorithms. The number of tests and the handling time for the tasks performed by laboratory personnel per batch of samples (the number of samples received per hour) were analyzed for both algorithms. The patients' diagnostic work flow was also analyzed for the time (in days) and the number of visits to the hospital required to obtain the final diagnostic results for the selected infection markers using the two screening algorithms.

## RESULTS

A total of 956 patients aged 18 to 82 years (mean age, 44.8 ± 18.1 years; 40.2% male) were enrolled in the study. The baseline characteristics of the patients included in the study are shown in Table 1. A total of 176 samples from the hospital repository of frozen samples were used to assess the sensitivity and the specificity of both algorithms for the detection of HIV-1/2 (Table 1). The sensitivity and specificity of the proposed algorithm with the Bio-Flash technology for the detection of anti-HIV-1/2 was 100%, whereas for the manual algorithm, the sensitivity was 80.0% and the specificity was 98.6% (Table 2). PPV and NPV were 100.0% using the algorithm with the Bio-Flash technology (Table 2). Eight samples with discordant results were observed, and the results were resolved by confirmatory Western blotting, in which six positive samples were confirmed to be negative and two negative samples were confirmed to be positive (Table 2).

**TABLE 1:** Baseline characteristics of the total patient population included in the study and the HIV-positive patient population^*a*^

Characteristic	Result for the following patients:				
	Total	HIV-1/2 positive	HCV positive	T. pallidum positive	HBsAg positive
No. (%) of patients	956 (100)	0 (0)	4 (0.41)	14 (14.6)	20 (2.09)
Age (yr)					
Range	18–82				
Mean ± SD	44.8 ± 18.1				
No. (%) of subjects of the following sex:					
Male	384 (40.2)	0 (0)	1 (25.0)	5 (35.7)	9 (45.0)
Female	572 (59.8)	0 (0)	3 (75.0)	9 (64.3)	11 (55.0)
HIV-positive patient population					
No. (%) of patients	176 (100)	24 (13.6)			
Mean ± SD age (yr)	45.5 ± 12.5				
No. (%) of subjects of the following sex:					
Male		11 (45.8)			
Female		13 (54.2)			

a HBsAg, hepatitis B virus surface antigen; HCV, hepatitis C virus; HIV, human immunodeficiency virus.

**TABLE 2:** Comparison of results obtained with the Bio-Flash technology-based and GICT screening algorithms with the clinical diagnosis for HIV detection using an additional panel of samples not included in the study^*a*^

Algorithm and result	No. of samples with the following clinical diagnosis^*b*^:		Sensitivity (%)	Specificity (%)	PPV (%)	NPV (%)
	Positive	Negative				
Bio-Flash technology-based screening algorithm (Bio-Flash anti-HIV 1+2)			100.0	100.0	100.0	100
Positive	24	0				
Negative	0	152				
GICT screening algorithm (Alere Determine HIV-1/2)			80.0	98.6	81.3	98.7
Positive	24	6				
Negative	2	144				

a Data are for 176 samples. HIV, human immunodeficiency virus; PPV, positive predictive value; NPV, negative predictive value.

b The clinical diagnosis was considered the gold standard for HIV detection.

The algorithm with the Bio-Flash technology showed a higher sensitivity for the detection of anti-HCV antibodies (100%) than the manual GICT screening algorithm (75.0%), with 100% specificity being found for both algorithms. PPV and NPV were 100.0% using the algorithm with the Bio-Flash technology (Table 3). One sample with discordant results was obtained using both algorithms, and the result was confirmed to be positive by Western blotting. Similarly, the automated algorithm with the Bio-Flash technology showed a higher sensitivity for the detection of HBsAg (100%) than the manual GICT screening algorithm (94.7%), with the specificity being 100% for both algorithms (Table 3). PPV and NPV were 100.0% using the algorithm with the Bio-Flash technology (Table 3). One sample with a discordant result was obtained using both algorithms, and the result was confirmed to be positive by testing for five different HBV infection markers (HBeAg, IgM anti-HBc, total anti-HBc, and anti-HBs) (Table 3). For T. pallidum detection, both algorithms were shown to be sensitive and specific (100% in all cases) (Table 3). PPV and NPV were 100.0% using both algorithms (Table 3), and no discordant results were observed.

**TABLE 3:** Comparison of Bio-Flash technology-based and GICT diagnostic algorithms for detection of markers of HIV, HCV, T. pallidum, and HBV infection^*a*^

Pathogen, algorithm, and result	No. of samples with the following clinical diagnosis^*b*^:		Sensitivity (%)	Specificity (%)	PPV (%)	NPV (%)
	Positive	Negative				
HIV-1/2						
Bio-Flash technology-based screening algorithm (Bio-Flash anti-HIV 1+2)			0	0	0	0
Positive	0	0				
Negative	0	956				
GICT screening algorithm (Alere Determine HIV-1/2)			0	0	0	0
Positive	0	0				
Negative	0	956				
HCV						
Bio-Flash technology-based screening algorithm (Bio-Flash anti-HCV)			100.0	100.0	100.0	100.0
Positive	4	0				
Negative	0	952				
GICT screening algorithm (HCV antibody strips; XiaMen)			75.0	100.0	100.0	99.9
Positive	3	0				
Negative	1	952				
T. pallidum						
Bio-Flash technology-based screening algorithm (Bio-Flash syphilis)			100.0	100.0	100.0	100.0
Positive	12	0				
Negative	0	942				
GICT screening algorithm (Alere Determine syphilis)			100.0	100.0	100.0	100.0
Positive	12	0				
Negative	0	942				
HBsAg						
Bio-Flash technology-based screening algorithm (Bio-Flash HBsAg)			100.0	100.0	100.0	100.0
Positive	20	0				
Negative	0	936				
GICT screening algorithm (HsBAg antibody detection; XiaMen)			94.7	100.0	100.0	99.9
Positive	18	0				
Negative	1	937				

a HBsAg, hepatitis B virus surface antigen; HBV, hepatitis B virus; HCV, hepatitis C virus; HIV, human immunodeficiency virus; NPV, negative predictive value; PPV, positive predictive value.

b The clinical diagnosis was considered the gold standard.

The level of consistency between the two algorithms for the detection of infection markers was high for all infections: anti-HIV-1/2 antibodies (κ value = 1), anti-HCV antibodies (κ value = 0.857), T. pallidum (κ value = 0.972), and HBsAg (κ value = 0.972) (Table 4). Using the algorithm with the Bio-Flash technology, a total of 909/956 patients were found to be negative for all infection markers at the early stages of screening (group A) and 47/956 were found to be positive. Of the 47 patients found to be positive, 31 participants were confirmed to be positive (group C) and 16 had a discordant diagnosis by use of the GICT screening algorithm. After analysis of the results by the confirmatory test, 14/16 participants were confirmed to be negative (group D) and 2/16 participants were confirmed to be positive (group E) (Fig. 2; Table 5). Using the manual GICT screening algorithm, a lower percentage of patients (896/956) was found to be negative for all infection markers at the early stages of screening (group A) and the 60 remaining patients were found to be positive. Of the 60 patients found to be positive, 31 were confirmed to be positive (group C) and 29 were confirmed to be negative (group B) by confirmatory tests (Fig. 2; Table 5).

**TABLE 4:** Level of consistency (κ value) between Bio-Flash technology-based and GICT screening algorithms for detection of markers of HIV, HCV, T. pallidum, and HBV infection^*a*^

Pathogen and GICT screening algorithm result	No. of samples with the following result by Bio-Flash technology-based screening algorithm:			κ value
	Positive	Negative	Total	
HIV-1/2				1.0
Positive	0	0	0	
Negative	0	956	956	
Total	0	956	956	
HCV				0.857
Positive	3	0	3	
Negative	1	951	952	
Total	4	951	955	
T. pallidum				0.972
Positive	12	0	12	
Negative	0	942	942	
Total	12	942	954	
HBsAg				0.972
Positive	18	0	18	
Negative	1	936	937	
Total	19	936	955	

a GICTs, gold immunoassays and colloidal tests; HBsAg, hepatitis B virus surface antigen; HBV, hepatitis B virus; HCV, hepatitis C virus; HIV, human immunodeficiency virus.

**TABLE 5:** Comparison of diagnostic characteristics between CLIAs with the Bio-Flash technology-based and GICT screening algorithms^*a*^

Patient group	Bio-Flash technology-based screening algorithm				GICT screening algorithm			
	% of pts	Time to results (h)	Day of final report	No. of hospital visits	% of pts	Time to results (h)	Day of final report	No. of hospital visits
A	95.08	1	1	1	93.72	1	1	1
B		1.5	1	1	3.03	24	2	2
C	3.24	2	1	1	3.24	24	2	2
D	1.46	24	2	2	0.00	48	6	2
E	0.21	24	2	2	0.00	48	6	2

a CLIAs, chemiluminescent immunoassays; GICTs, gold immunoassays and colloidal tests; pts, patients.

**FIG 3:**
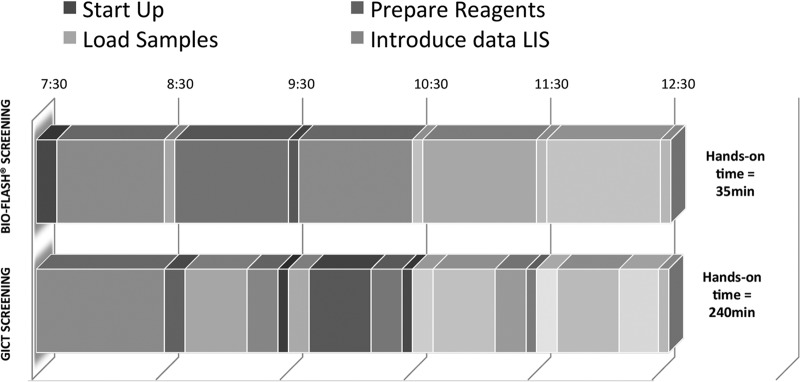
Hands-on time per day using the automated chemiluminescent Bio-Flash technology-based screening algorithm (the new proposed laboratory procedure) and the manual GCIT screening algorithm (the regular laboratory procedure).

Using the new algorithm with two Bio-Flash instruments, handling was required only to load the sample batches every hour. The first set of results was generated within 30 min of loading of the samples, and continuous loading of the samples gave the results for the four markers of infection from 30 samples in 60 min. The total hands-on time required with the new automated algorithm with the Bio-Flash technology was 35 min for complete screening analysis, including start-up and maintenance, whereas the manual GICT screening algorithm required 240 min for the generation of the final report (Fig. 3).

Use of the automated Bio-Flash technology-based screening algorithm also reduced the time required to obtain complete diagnostic test results for the infection markers and the frequency of hospital visits for patients compared with the time required by use of the regular laboratory procedure with the manual GCIT screening algorithm (Table 5). Use of the algorithm with the Bio-Flash technology required a maximum of 2 days and a maximum of two hospital visits to reach a final diagnosis for all groups, whereas use of the manual GICT screening algorithm required 6 days to reach a final diagnosis for some patients (Fig. 2). Complete diagnostic results were obtained for 95.08% of the patients as early as 60 min after the collection of blood samples, 98.32% of patients received the complete diagnostic result on day 1 (groups A, B, and C), and only 1.67% of patients were required to schedule a second visit (Table 5) when the algorithm with Bio-Flash technology was used. With the manual GCIT screening algorithm, 93.72% of patients received complete diagnostic results within 60 min on day 1 (group A), while the rest of the patients (6.27%; groups B and C) were required to schedule a second visit to the hospital and received their results on day 2 (Table 5); no patients were included in groups D and E.

## DISCUSSION

The present study compared the sensitivity, specificity, and work flow characteristics of the automated two-step Bio-Flash technology with those of the manual GICT screening algorithm for screening for markers of infection in patients undergoing GI endoscopy. The Bio-Flash technology-based screening algorithm showed 100% sensitivity and specificity for the detection of markers of HIV-1/2, HCV, HBsAg, and T. pallidum infection. The newly proposed screening algorithm with the Bio-Flash technology also reduced the handling time required for the assay, the overall time required to generate diagnostic results, and the number of hospital visits compared with the results obtained with the routinely used manual GICT screening algorithm for the detection of infection markers in these patients.

Automated CLIA analyzers are used for routine serological assays in high-volume clinical laboratories. These instruments offer excellent precision and reliability, high-speed throughput, random access, and technical simplicity. Although automated CLIAs are gradually replacing EIAs, data from published studies have compared the results obtained with algorithms that use the two techniques (9, 10, 12, 15–17). It has been reported that the detection of infection markers requires all screening assays to have high levels of sensitivity and specificity (9–11, 18). The present study demonstrated for the first time that the use of an algorithm based on automated CLIAs before GI endoscopy increased the sensitivity of detection of HIV, HCV, and HBV, increased the specificity of detection of HIV, and maintained the specificity of detection of HCV, HBV, and T. pallidum, while it allowed a substantial reduction in the hands-on time and the total time required to obtain the diagnostic test results. These results are in line with those of previous studies, where the use of CLIAs was reported to be highly specific and sensitive for the diagnosis of HCV and HBV infection compared with conventional EIAs (9, 10, 12).

It was also reported in the present study that the algorithm with the Bio-Flash technology showed an increased specificity for the detection of HIV due to the observation of a lower number of false-positive results. False-positive results for HIV-infected samples are problematic, and it is important to use approaches that minimize the number of biological false-positive screening test results. Since biological false-positive results occur for a variety of reasons, confirmatory tests are necessary during screening. It is generally recommended by assay manufacturers and health authorities that for all positive samples serological screening be repeated twice using the same assay before proceeding to confirmatory tests for these repeatedly reactive samples (11, 19–21). Assays that use the Bio-Flash technology have been shown to be useful for the detection of infection markers, although more research about their implementation, acceptability, and costs in routine clinical practice is needed (22).

An important advantage of the proposed algorithm with the automated Bio-Flash technology reported in the present study was the reduction in the working time of the laboratory technicians. The results of the study indicated that the automated Bio-Flash technology-based screening algorithm reduced the operation time of the laboratory technicians by 85% (205 min) per day for the detection of markers of infection in patients before GI endoscopy, saving up to 24 h/week. Further, the use of the proposed algorithms also improved the laboratory work flow in terms of connection with the laboratory information system (LIS), the availability of historic results, interpretation of results, and automation. Importantly, the proposed algorithm also increased the percentage of patients receiving their final report and confirming their GI surgery on the same day that the blood sample was collected, avoiding the need for a second visit to the hospital. Thus, the use of this algorithm can help with the early detection and management of an infectious disease in case of positive results.

Finally, due to its increased sensitivity and specificity, use of the proposed Bio-Flash technology-based screening algorithm reduced the number of samples for which the results needed to be confirmed in an external laboratory. Of note, in this study, no patients were included in groups D and E of the manual GICT screening algorithm; a confirmatory Western blot analysis would be needed for patients in these groups. Data documented in the Department of Blood Transfusion, General Hospital of Beijing Military Region, showed that a total of 82 samples (*n* = 12 samples positive for HCV, *n* = 29 samples positive for T. pallidum, and *n* = 41 samples positive for HIV) in 2015 and 76 samples in 2014 (*n* = 13 samples positive for HCV, *n* = 7 samples positive for T. pallidum, and *n* = 56 samples positive for HIV) were sent to an external laboratory for confirmatory Western blot assay, accounting for 0.38% and 0.30% of all specimens, respectively. Thus, the use of the currently proposed automated Bio-Flash technology may be beneficial in the processing of these samples.

In conclusion, the present study demonstrated for the first time the advantages of a screening algorithm based on the automated Bio-Flash technology for the detection of HIV, HCV, HBV, and syphilis in patients undergoing GI endoscopy.
